# Necrotizing Sialometaplasia and Bulimia: A Case Report

**DOI:** 10.3390/medicina56040188

**Published:** 2020-04-19

**Authors:** Francisco Salvado, Miguel de Araújo Nobre, João Gomes, Paulo Maia

**Affiliations:** 1 University Clinic of Stomatology, Faculty of Medicine, University of Lisbon, 1649-028 Lisbon, Portugal; fjsalvado2002@yahoo.com; 2 Egas Moniz University Institute, 2829-511 Caparica, Portugal; jcg@egasmoniz.edu.pt (J.G.); paulormaia@gmail.com (P.M.); 3 Research and Development Department, Maló Clinic, 1600-042 Lisbon, Portugal; 4 Faculty of Dental Medicine, Catalunya International University, 08195 Barcelona, Spain

**Keywords:** necrotizing sialometaplasia, bulimia, bulimia nervosa, diagnosis, epidemiology

## Abstract

Bulimia is an eating disorder with a great prevalence in young women. Due to its multifactor ethiology, bulimia has systemic consequences. In the literature, necrotising sialometaplasia is seldom associated with bulimia. Its etiopathogenesis is discussed by several authors; nevertheless, the consensus does not consider the relevance of local trauma associated with induced vomiting. A case of necrotising sialomethaplasia, presented with a single hard palatal ulcer in a bulimic woman is described in the present report. The patient did not present significant systemic laboratorial values, nor physical weight variations, which highlights the relevance of performing a complete medical clinical history when diagnosing this rare pathology.

## 1. Introduction

Necrotizing sialometaplasia (NS) is a rare pathology with a benign behavioral nature, which affects the salivary glands. It is generally more frequent in men in the fourth decade of life, with a reported average age of 49 years for men and 46 years for women (based on figures for men and women between 15 and 79 years of age) [1].

The etiopathogenesis of NS is somewhat controversial: it appears neither to be an intrinsic pathology of the salivary gland tissue, nor the manifestation of a specific systemic condition. Despite the possibility of it occurring spontaneously, local irritative or traumatic factors such as smoking, alcohol abuse, drugs and prosthetic trauma seem to be directly involved in a significant number of cases [2,3].

Regarding its histological appearance, it is considered, in general, that systemic diseases with vascular compromising effects may also be associated with NS; diabetes, atherosclerosis, drepanocytosis, hypertension and Buerger’s disease all induce vascular alterations, ischemically compromising some organs or anatomical areas of the human body, which is why they are associated with NS lesions [4,5]. Some iatrogenic forms, secondary to local anesthesia, endotracheal intubation and dental extractions, have also been described in the literature [6]. A common characteristic in all cases of acute NS after local anesthesia was the fact that vasoconstrictors were always used, which seems to confirm the relevance of vascular alterations in this pathology [7]. Physical and chemical irritation of the oral mucosa apparently plays an important role in triggering NS, since very hot foods, tobacco and alcohol consumption are present in a large number of cases of this pathology [8].

Previously, NS was associated with bulimia and anorexia [2,9,10]. While the association of NS with anorexia needs to be properly disclosed, the association with bulimia seems plausible from an epidemiological point of view, since the incidence of this pathology is accompanied by significant vascular changes. Generally, the clinical manifestations of NS are characterized by palatine ulcerations. The base of the lesion is filled with necrotic tissue and a whitish leucocyte fibrin exude. It is common for the underlying bone to be exposed without significant osteolysis. This characteristic is not, however, pathognomonic of the disease, as some authors have registered NS episodes with the complete destruction of the palatine arch bone and presence of an oroantral fistula [11]. The ulceration has well-defined hemorrhagic limits that are more often elongated in the anterior–posterior direction. The edges are protruding and appear to be in the healing phase, presenting an inversion of the superficial mucosa to the lesions’ internal aspect. In the periphery, an erythematous or whitish halo can be identified. In about 25% of cases, during the initial phases, this lesion appears as a hard swelling adherent to the deep planes, with a whitish or erythematous surface and numerous dilated vessels, evolving to ulceration [12]. Previous authors registered cases that only present as tumor forms without evolving to ulceration, without pain, nor with local neurological alterations. These are likely sub-acute manifestations of the disease that are possible to be positively influenced by early therapy with wide-spectrum antibiotics [13].

A single lesion in the transition between the hard and soft palate is the most frequent presentation, although bilateral and even multiple lesions with random distribution on the hard palate may appear [3]. The palatal location is not universal. Any zone with ancillary salivary glands can be affected; nevertheless, more rarely, the main salivary glands may also be affected. Exceptionally, the condition may also affect the seromucosal glands of the upper airways (nasal cavities, sinus cavities and nasopharynx) [14,15].

Pain is the most frequent symptom, although cases presenting local paresthesia, local anesthesia or cases that are completely asymptomatic are also described. Pain intensity does not appear to be related to the morphological severity of the lesion [16].

Differential diagnosis should take into account the two clinical presentations of NS. The tumor form can be confused with periodontal abscesses or tumors of the minor salivary glands such as pleomorphic adenoma, cystic adenoid carcinoma and epidermoid mucus carcinoma, which are more frequent pathologies than NS. The ulcerated form may resemble malignant tumors in the same location (salivary glands or mucosa), major oral ulcerations or rarer pathologies, such as syphilitic or tuberculosis ulcerations [17,18].

The name necrotizing sialometaplasia reflects the histological aspect of the lesion. The anatomical–pathological diagnosis follows five main criteria: ischemia or lobular necrosis, conservation of the nucleus morphology of epithelial cells, metaplasia involving both mucous canals and acini, presence of inflammatory infiltration and granulation tissue and, finally, conservation of the lobular aspect, even though inflammatory and metaplastic changes reach multiple glandular lobes [19]. It should be noted that, in non-ulcerated forms, the surface epithelium is maintained. In the healing phase, the regression of both inflammatory infiltration and ulceration aspects and progressive reepithelization are observed. NS leaves, as sequelae, a limited decrease in the number of acini and canals, with the appearance of scar tissue associated with fibrosis and adipocytes [20].

As a rule, NS has a limited self-evolution, not requiring aggressive therapeutic measures. Enhanced local hygiene measures should be used during the ulcerated phase of the disease [21]. When pain is part of the clinical symptoms, systemic analgesics such as paracetamol should be used. The application of local anesthetics on the ulcerated zone before meals should be carefully monitored to prevent local trauma with food.

Ulcerated lesions heal without leaving significant scars, although, in some cases, a small depression, in tandem with pre-existing ulceration, may be observed. The disease is not recurrent.

As mentioned by the authors, the importance of a correct diagnosis of NS lies in the fact that it can be confused with a malignant tumor whose treatment, which is more aggressive, can leave significant sequelae.

## 2. Case Report

A 22-year-old female patient, followed by the Psychiatric Department for Bulimia, presented at a stomatology appointment for unpainful oral ulceration (15 mm × 7 mm) in the central palatine region that had lasted for 3 weeks. She reported that she was orphaned and adopted at 3 years old. Her physical aspect was compatible with her real age without alterations, and her mucosa appeared slightly pale. The patient stated that, in the initial phase, the lesion was a small non-painful ulceration that had increased in volume in the past two weeks. The patient denied experiencing systemic symptomatology in particular: fever, marked weight loss, joint pain, nor the ingestion of medicines or recognized contact with contagious infectious patients. The patient declared a one-year history of increased food intake followed by vomit provocation, with a loss of 10 kg during this period.

**Figure 1:**
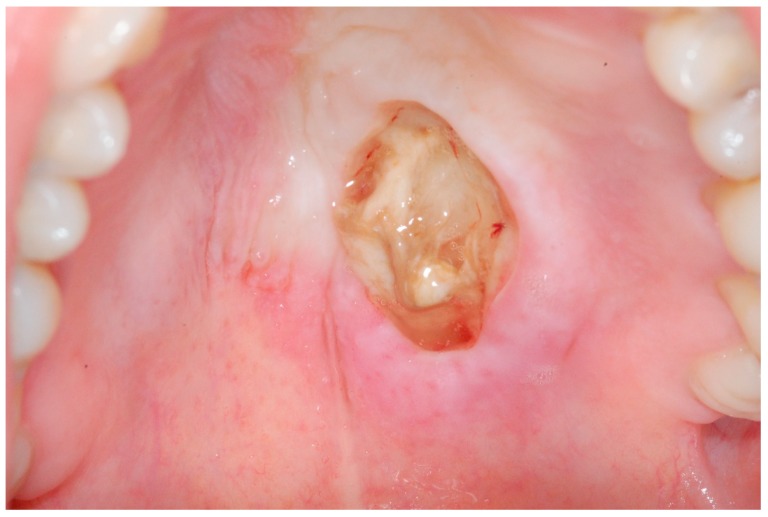
Intra-oral photograph of the palatal aspect exhibiting the lesion. Note the edges of the lesion with well-defined limits and the erythematous halo with well-defined limits surrounding the whole lesion.

During clinical examination, a unique crateriform ulcerated lesion, localized centrally on the hard palate (next to the transition to the soft palate), which was about 8 mm in diameter (anterior–posterior) was observed. The base of the lesion was filled with whitish necrotic remains, with no exposure of the underlying bone. The edges of the lesion had well-defined limits, a hard consistency and were slightly hemorrhagic upon contact. Surrounding the entire lesion, an erythematous halo with well-defined limits was identified (Figure 1).

**Figure 2:**
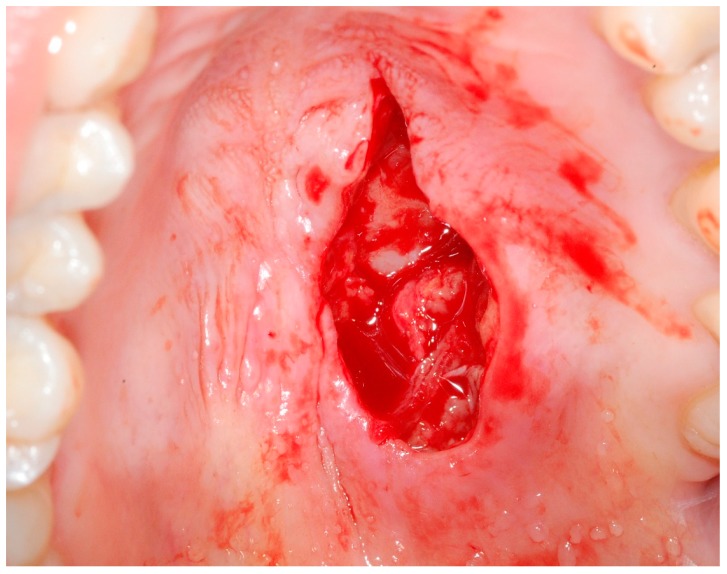
Intra-oral photograph immediately after performing the incisional biopsy of the lesion.

**Figure 3:**
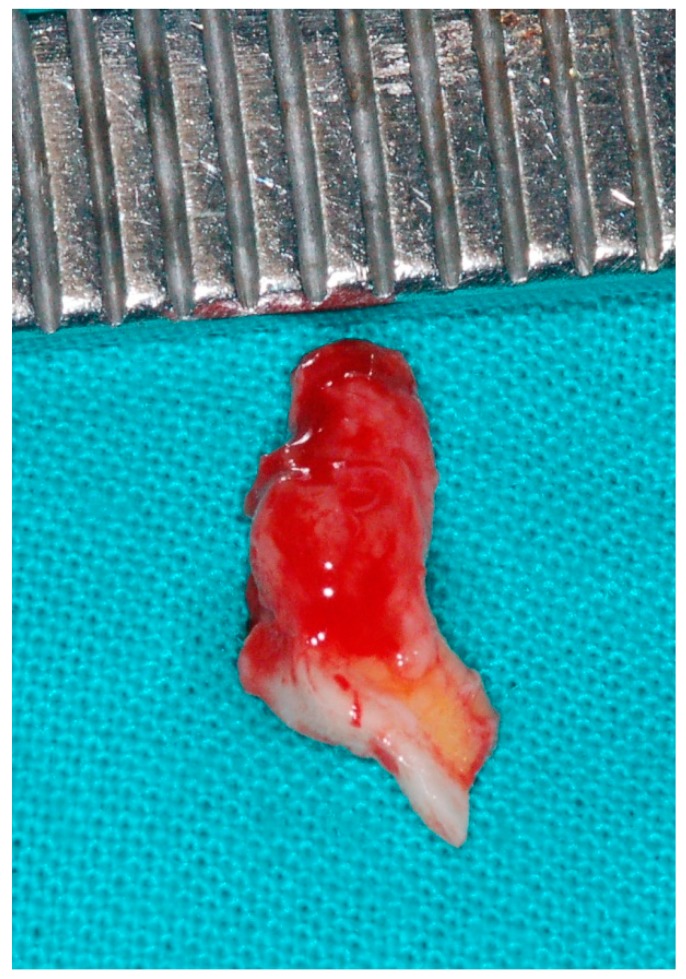
Photograph immediately after performing the incisional biopsy of the lesion. Note that the biopsy involved both the edges of the lesion, a portion of tissue from the central area of the lesion and healthy tissue of the palate.

Laboratory tests that were performed to investigate hematological (anemia) or immunological alterations provided negative results. Human immunodeficiency virus (HIV) testing was also negative. The patient’s C-reactive protein levels and Erythrocyte Sedimentation Rate were slightly elevated. The diagnostic hypotheses considered were necrotizing sialometaplasia vs. an ulcerated tumor of the minor salivary glands vs. a major mucosal ulceration. An incisional biopsy of the lesion was performed covering the most anterior area of the ulceration, involving the edges and healthy tissue of the palate and including a portion of tissue from the central area of the lesion (Figure 2 and Figure 3). 

The postoperative period occurred without complications and without aggravation of the clinical characteristics of the lesion.

**Figure 4:**
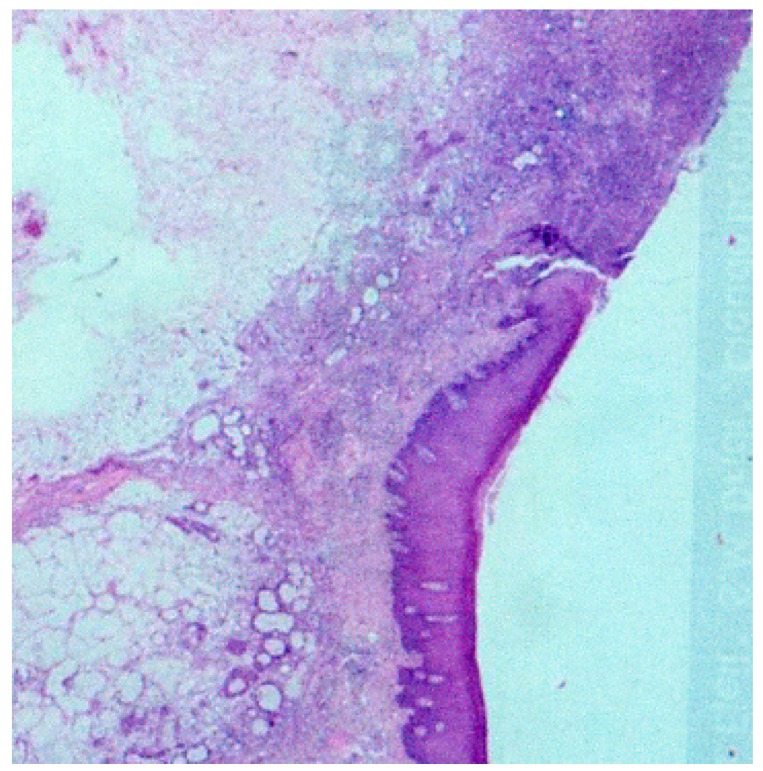
Biopsy exhibiting a slightly hyperplastic epithelium, limiting the ulceration surface, with both the foci of necrosis and some minor salivary glands in pre-necrotic status in the underlying connective tissue.

**Figure 5:**
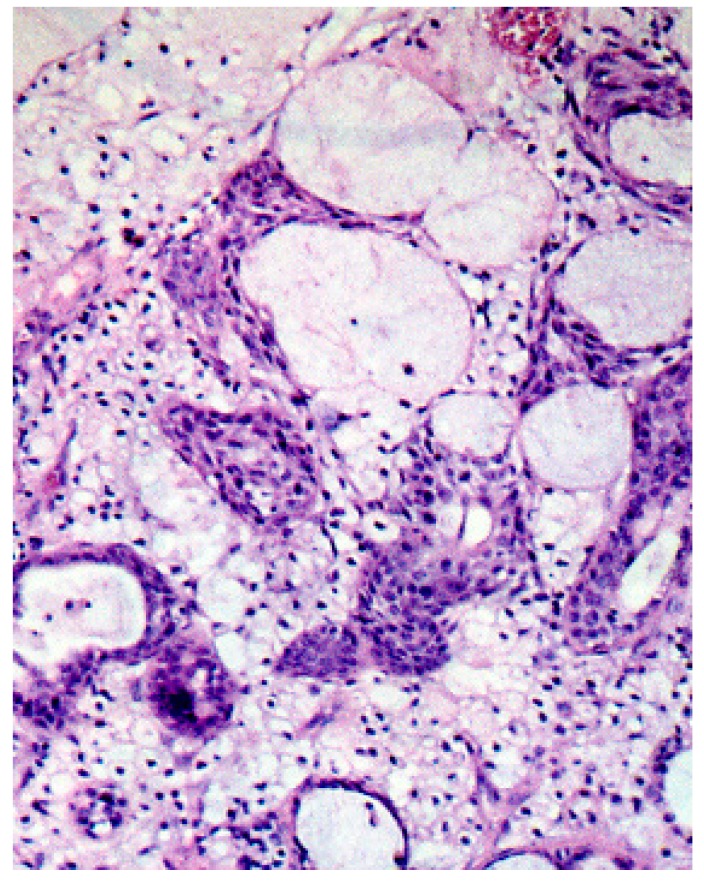
Magnification of the biopsy exhibiting necrosis and inflammatory infiltration of acinar cells, foci of malpighian metaplasia in residual channels and some acini in canalicular transformation.

The biopsy exhibited a slightly hyperplastic epithelium, limiting the ulceration surface. In the underlying connective tissue, we observed the foci of the necrosis and some minor salivary glands in pre-necrotic status. Most acinar cells exhibited absent cores and the presence of vacuoles in the cytoplasm, while maintaining their lobular architecture. At greater magnifications, the necrosis and inflammatory infiltration of acinar cells, the foci of malpighian metaplasia in the residual channels and some acini in canalicular transformation were confirmed (Figure 4 and Figure 5).

These histological findings were characteristic of necrotizing sialometaplasia. Pain treatment was performed with paracetamol if needed. Adequate oral hygiene (tooth brushing and chlorhexidine rinses) was prescribed to prevent the superinfection of the ulcerated area. 

**Figure 6:**
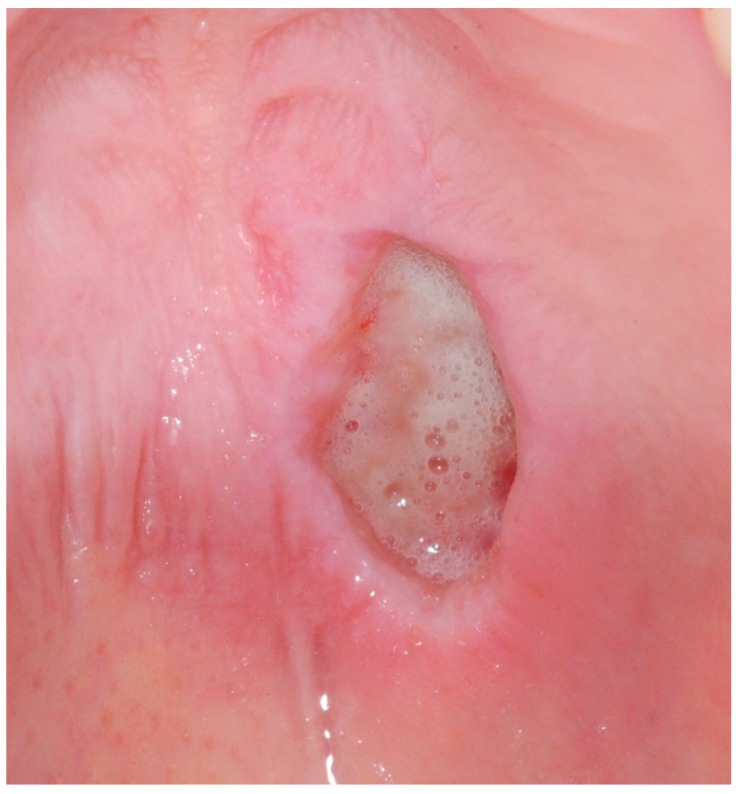
Intra-oral photograph of the palatal aspect four weeks post-operatively. Note the improvement in the lesion.

**Figure 7:**
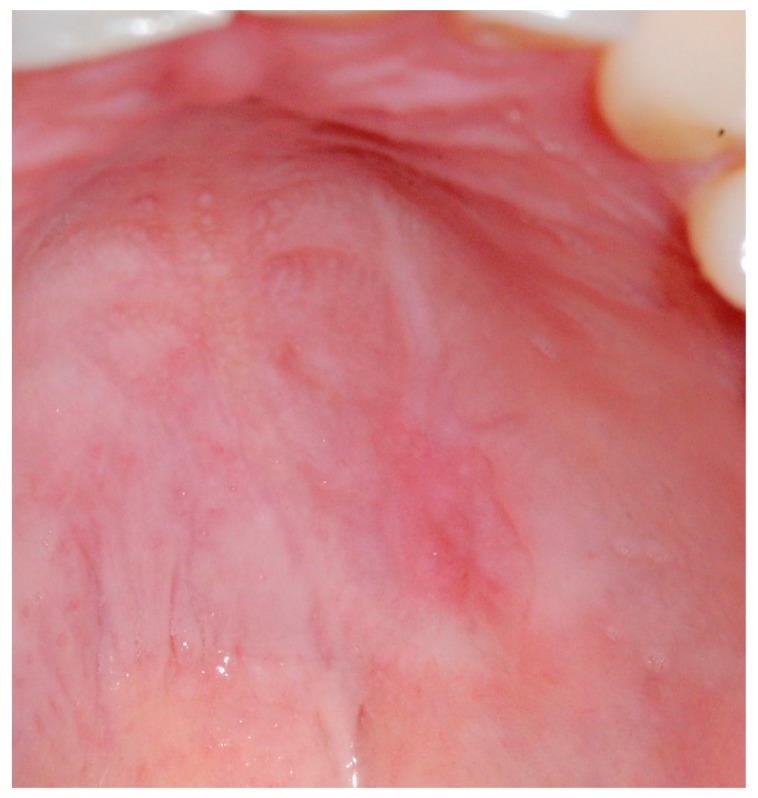
Complete healing of the lesion after 8 weeks.

The patient performed periodic controls every two weeks. After four weeks, a marked improvement in the lesion was observed (Figure 6) and, at the end of 8 weeks, the lesion was completely healed (Figure 7).

## 3. Discussion

Bulimia can be considered an eating disorder [22] (International Classification of Diseases 11th Revision Code: 6B81 [23]). Characteristically, these patients ingest a large amount of food over a short period of time. This type of feeding is accompanied by fear of increasing weight. This fear causes the large food intake to be alternated with periods when the patient stimulates vomiting in order to prevent the digestion cycle. At the same time, these patients usually increase their physical exercise, ingest aggressive laxatives, diuretics or other appetite-inhibiting medications. A misconception that this type of pathology is present mainly in middle or upper class young women is widespread. In fact, this disease is more frequent in women during puberty or right after this period, but can occur in both genders and at all ages [24].

Potential contributing factors for the development of bulimia include: stress, culture, family history of the disease and depression [25]. Some authors report that genetic factors may also predispose a person to bulimia [26]. A bulimia patient may present as thin, average or obese, making primary and early recognition of the disease challenging. Authors report that bulimia is a variant of anorexia nervosa, concluding that bulimia may be an earlier form of this disease [27]. There are oral alterations that may increase the suspicion of a patient with bulimia, including larger jugal regions, signs of trauma in the jugal region, perimylolysis, dental erosion, dental hypersensitivity and increased visible vascularization of the entire oral mucosa; all these signs can be observed in patients with recurrent vomiting [10,28,29].

The etiology of necrotizing sialometaplasia may be related to systemic diseases, particularly those with significant vascular alterations, including diabetes, atherosclerosis and drepanocytosis [21,30]. Changes in appetite, such as those caused by anorexia nervosa, can cause vascular alterations, such as fragility in the walls of blood cells, contributing to the appearance of NS.

Clinical cases of bulimia associated with necrotizing sialometaplasia are not frequent in the scientific literature. In bulimic patients, where weight changes are not dramatic (as was the case with the present patient), vascular frailty is not significant and therefore should not be included, per se, in the vascular etiologies of NS [31]. These patients cause vomiting by the introduction of their fingers into the oral cavity, sometimes in a violent and repeated way. This action causes trauma of the palate, leading to the appearance of lesions. On the other hand, the violent movement of vomiting itself can cause vascular micro ruptures, with micro ischemia in the stroma of the minor salivary glands. Moreover, lesions associated with bulimia are present in the ulcerated form. Many of these patients receive antidepressant therapy, which can cause xerostomia and the fragility of the oral mucosa [32], which can contribute to greater vascular sensitivity to local trauma. Endotracheal intubation and hard food consumption were also reported by some authors as etiological factors of intraoral tissue trauma [33]. Therefore, due to all these factors, bulimia should be considered in the process of etiological diagnosis of NS.
